# One-Step Process for Environment-Friendly Preparation of Agar Oligosaccharides From *Gracilaria lemaneiformis* by the Action of *Flammeovirga* sp. OC4

**DOI:** 10.3389/fmicb.2019.00724

**Published:** 2019-04-17

**Authors:** Xinglin Chen, Li Li, Zhuhua Chan, Runying Zeng, Mengshi Lin, Hetong Lin

**Affiliations:** ^1^College of Food Science, Fujian Agriculture and Forestry University, Fuzhou, China; ^2^State Key Laboratory Breeding Base of Marine Genetic Resource, Third Institute of Oceanography, Ministry of Natural Resources, Xiamen, China; ^3^Food Science Program, Division of Food System and Bioengineering, College of Agriculture, Food and Natural Resources, University of Missouri, Columbia, MO, United States

**Keywords:** *Gracilaria lemaneiformis*, oligosaccharides, *Flammeovirga* sp. OC4, one-step process, response surface methodology, optimization

## Abstract

Oligosaccharides extracted from agar *Gracilaria lemaneiformis* (*G. lemaneiformis*) have stronger physiological activities and a higher value than agar itself, but the pollution caused by the extraction process greatly restricts the sustainable use of agar. In this study, four bacterial strains with a high ability to degrade *G. lemaneiformis* were isolated from seawater by *in situ* enrichment in the deep sea. Among them, *Flammeovirga* sp. OC4, identified by morphological observation and its 16S rRNA sequencing (98.07% similarity to type strain JL-4 of *Flammeovirga aprica*), was selected. The optimum temperature and pH of crude enzyme produced by *Flammeovirga* sp. OC4 were 50°C and 8, respectively. More than 60% of the maximum enzyme activity remained after storage at pH 5.0–10.0 for 60 min. Both Mn^2+^ and Ba^2+^ could enhance the enzyme activity. A “one-step process” for preparation of oligosaccharides from *G. lemaneiformis* was established using *Flammeovirga* sp. OC4. After optimization of the Plackett–Burman (PB) design and response surface methodology (RSM), the yield of oligosaccharides was increased by 36.1% from 2.71 to 3.09 g L^−1^ in a 250-mL fermenter with optimized parameters: 30 g L^−1^ *G. lemaneiformis* powder, 4.84 g L^−1^ (NH4)_2_SO_4_, 44.8-mL working medium volume at 36.7°C, and a shaking speed of 200 × *g* for 42 h. The extracted oligosaccharides were identified by thin layer chromatography (TLC) and ion chromatography, which consisted of neoagarobiose, agarotriose, neoagarotetraose, agaropentaose, and neoagarohexaose. These results provided an alternative approach for environment-friendly and sustainable utilization of algae.

## Introduction

Oligosaccharides are carbohydrates that contain 2–20 sugar units joined by glycoside bonds. Some oligosaccharides are biologically active substances that possess antimicrobial activity, immunoenhancing ability, antitumor activity (Fang et al., 2012; Zou et al., 2016; Chen et al., 2017), and antioxidant activity (Chen et al., 2015; Malunga and Beta, 2015; Hamley-Bennett et al., 2016) and have great potential in nutrition (Kashtanova et al., 2016; Marcotuli et al., 2016), health care (Mensink et al., 2015; Zhang et al., 2016; Di et al., 2017), disease diagnosis, and control (Kazłowski et al., 2012; Zhang et al., 2016). To date, oligosaccharides derived from plants and chitins have been used widely (Wojtkowiak et al., 2013; Hamley-Bennett et al., 2016; Yin et al., 2016). On the other hand, algae, an inexpensive and abundant biomass, is attracting increasing attention because it can be used for the production of algae oligosaccharides that have better physiological activity and a higher value than algae polysaccharides (such as agar, carrageenan, and alginate). However, current studies are mostly focused on agarase (Kim et al., 2017, 2018; Qu et al., 2018; Schultz-Johansen et al., 2018), and little has been done in the direct and efficient preparation process of algae oligosaccharides from algae. Currently, algae oligosaccharides are prepared by a two-step process: step 1 is the extraction of algae polysaccharides from algae, which consumes a lot of strong acids and alkalis, diatomite and perlite, and produces useless algal dregs; step 2 is the degradation of polysaccharides by expensive agarases or strong acids, which can result in great pollution to the environment. However, with growing ecological awareness, the preparation process with high pollution has raised concerns in the food industry and is becoming a major obstacle limiting the sustainable use of algae oligosaccharides. Thus, there is a need to develop a more efficient and environment-friendly approach of direct enzymatic degradation of algae for oligosaccharide production, which is also named “one-step process.”

To establish a “one-step process,” suitable agarolytic bacterial strains that can produce enzymes should be screened first. The deep sea environment harbors a massive pool of biodiversity, which is a good source for agarolytic bacterial strains (Kamjam et al., 2017; Huo et al., 2018). Because of extreme ecological environment such as high pressure (Case et al., 2017), high or low temperature (Mino et al., 2013), and oligotrophic environment (Picard and Ferdelman, 2011; Sohlberg et al., 2015), deep sea microorganisms can form unique physiological structures and metabolic mechanisms, which are excellent sources for acquiring the strain with an inventory of enzymes that can be used for producing oligosaccharides directly from algae.

Fermentation conditions of the deep sea strains are important parameters for biotransformation to oligosaccharides, which should be optimized to achieve higher productivity of oligosaccharides. Several techniques have widely been used to increase the production of oligosaccharides, including the experimental–statistical method (Dotsenko et al., 2016; Sen et al., 2016), the two-level Plackett–Burman (PB) design (Purama and Goyal, 2008; Zhou et al., 2014), and the response surface methodology (RSM) (Yoshida et al., 2010; Vega and Zúniga-Hansen, 2011).

In this study, some agarolytic bacterial strains were isolated from the deep sea through *in situ* enrichment. *Gracilaria lemaneiformis* (*G. lemaneiformis*) was used as the sole carbon source. One of these isolated agarolytic strains was selected to produce oligosaccharides by degrading *G. lemaneiformis*. PB design was used to investigate the factors affecting the production of oligosaccharides, and the RSM method was used to further optimize the parameters of biotransformation to oligosaccharides from *G. lemaneiformis*. The objectives of this study were to provide a theoretical basis for producing algae oligosaccharides from *G. lemaneiformis* with less pollution to the environment and to provide algae oligosaccharides for potential use in agro-food systems and in the nutraceutical industries.

## Materials and Methods

### *In situ* Enrichment in the Deep Sea

*Gracilaria lemaneiformis* was washed by tap water and spin dried, which was repeated three times, and then air dried. The sample was then cut 2 or 3 cm in length and ground to powder for further use. The deep sea strains were enriched using a special *in situ* sampling equipment, which was loaded with *G. lemaneiformis* powders, and placed at the sampling site in the sea water with a depth of 2,000 m, 21°03′N, 118°23′E of the South China Sea for 1 year (Supplementary Figure S1).

### Screening and Identification of Agarolytic Strains

The enriched samples were transferred onto the plate of the basic culture medium (BCM) that contained the following components (g L^−1^): *G. lemaneiformis* powder, 30; peptone, 5; (NH_4_)_2_SO_4_, 4; KCl, 10. Strains with a transparent ring were acquired and purified. The optimal temperature and relative activity of the crude enzyme of the strains were investigated by shake flask experiments. The single colony with a transparent circle was selected and transferred to another BCM plate and cultured at 28°C. The newly grown strains were observed and photographed under a transmission electron microscope (TEM). A bacterial genomic DNA extraction kit (Sangon Biotech. Co., Ltd., Shanghai, China) was used to extract the genomic DNA of the strain as a template for PCR amplification of 16S rRNA with an upstream primer (27F: 5′-AGAGTTTGATCCTGGCTCAG-3′) and a reverse primer (1492R: 5′-GGTTACCTTGTTACGACTT-3′) (Delong, 1992) with the following program: 94°C for 4 min (94°C for 30 s, 55°C for 30 s, 72°C for 60 s, repeated 30 times); 72°C for 5 min. Phylogenetic analysis was performed using MEGA version 5.1 after multiple alignment of data using DNAMAN version 7.0.

### Characterization of Crude Agarase Enzyme

The strain mentioned above was cultured in BCM by shaking at 28°C for 36 h and then centrifuged at 13,000 × *g* for 10 min to acquire the supernatant as crude enzyme solution, whose activity was tested by detecting the release of oligosaccharides according to the 3,5-dinitrosalicylic acid (DNS) method (Hou et al., 2015; Chen et al., 2016).

The effect of temperature was studied by measuring the enzymatic activity at several temperatures ranging from 30 to 80°C at pH 7.4 of phosphate-buffered saline (PBS) buffer. Meanwhile, crude enzyme in pH 7.4 of PBS buffer without substrate was incubated for a period of time (0, 48, 74, 99, 120, and 144 h) at several temperatures (30, 40, and 50°C) to investigate the effect of thermostability by measuring the remaining enzyme activity.

The effect of pH was investigated by measuring the crude enzyme activity at 50°C in several pH values ranging from pH 3.0 to 11.0 in the buffers with the concentration of 50 mM: Na_2_HPO_4_/citric acid solution (pH 3.0–8.0), Tris-HCl buffer (pH 7.0–9.0), and Gly/NaOH buffer (pH 9.0–11.0). Meanwhile, the effect of pH stability was evaluated by measuring the remaining activity of the crude enzyme preincubated at 50°C for 60 min in several solutions from pH 3.0 to 11.0.

The effects of the chelators and metal ions were studied by detecting the enzyme activity at 10 mM of ethylene diamine tetraacetic acid (EDTA) or different metal ions, which were listed below: chelator (EDTA) and metal ions (Co^2+^, Mg^2+^, Cu^2+^, Na^+^, Ca^2+^, Mn^2+^, K^+^, Fe^2+^, Ba^2+^, Sr^2+^, and Zn^2+^). All the above-mentioned experiments were conducted in triplicate.

### Single Factor Optimization Experiments

Seven experimental groups were set with concentration of *G. lemaneiformis* from 10 to 40 g L^−1^, five values per interval. Other factors were exactly the same as the BCM condition.

Nitrogen sources, which were mainly used in the synthesis of metabolites and cell substances, could be divided into inorganic nitrogen sources or nitrogen sources. KNO_3_, NaNO_3_, (NH_4_)_2_SO_4_, NH_4_NO_3_ (5 g L^−1^; inorganic nitrogen sources) and yeast extract, peptone, beef extract, bean pulp (4 g L^−1^; organic nitrogen sources) were evaluated to determine the best one. Each group was a BCM containing this type of nitrogen source instead of the original one. After that, different concentrations of the best nitrogen sources were compared to determine the optimal concentration.

A solution of different inorganic salts (NaCl, KCl, MgCl_2_, and MgSO_4_) was prepared with distilled water to a final concentration of 10 g L^−1^. The oligosaccharide products in these solution and seawater were compared to select the best one, following concentration screening from 5 to 30 g L^−1^.

Based on the BCM condition, different temperatures (25, 28, 30, 33, 37, and 40°C), initial pH (5, 6, 7, 8, 9), inocula (1%, 3%, 5%, 8%, and 10%), and medium volume (30, 40, 50, 60, and 70 mL) were carried out separately.

### Experimental Design for Oligosaccharide Production

Since every single variable mentioned above was obtained under certain conditions, the PB design was used to screen significant variables that influence the oligosaccharide production among all the medium components. N variables could be evaluated in N+1 experiment by PB design and examined at two levels: +1 for a high level, and −1 for a low level. In this study, these variables were selected: peptone, KCl, (NH_4_)_2_SO_4_, initial pH, temperature, 250-mL Erlenmeyer flask with a working medium volume, and inocula. The factors and the levels of each factor are illustrated in Table 1.

**Table 1:** Experimental variables at different levels for oligosaccharide production by *Flammeovirga* sp. OC4 using the PB design.

Symbol code	Variables	Units	Level	
			−1	+1
A	Peptone	g L^−1^	2	5
B	KCl	g L^−1^	10	15
C	Dummy	–	–	–
D	(NH_4_)_2_SO_4_	g L^−1^	2	6
E	Initial pH	–	7	8
F	Dummy	–	–	–
G	Inocula	% (*v*/*v*)	3	5
H	Medium volume	mL	40	60
J	Dummy	–	–	–
K	Temperature	°C	33	37
L	Dummy	–	–	–

*PB, Plackett–Burman.*

Response surface methodology was used for optimizing the screened variables to increase the yield of oligosaccharides from *G. lemaneiformis* powders by the action of *Flammeovirga* sp. OC4. Developed by Design Expert 8.0.6, central composite design (CCD) under RSM was used for optimizing the concentrations of the significant factors and determining a total of 20 experimental runs with six replicated center points. Each factor was investigated in five levels (+1.682, +1, 0, −1, and −1.682). Each of the factors was calculated according to Eq. (1).

(1) $$x_{i}=(X_{i}-X_{0})/\Delta X_{i},\;i=1,\;2,\;3,\;\ldots ,\;k$$

where *x_i_* and *X_i_* are the codified and actual values, respectively. *X*_0_ is the value of *X_i_* at the center point, and Δ*X_i_* is the step change value. The relationship between the dependent and independent variables was explicated by the second-order polynomial Eq. (2) below.

(2) $$Y=\beta_{0}+\Sigma \beta_{i}x_{i}+\Sigma \beta_{ii}x_{i}{}^{2}+\Sigma \beta_{ij}x_{i}x_{j},\;i=1,\;2,\;3,\;\ldots ,\;k$$

where *Y* is the predicted response, *x_i_* and *x_j_* are the coded independent factors that influence the response variable *Y*, β_0_ is the intercept, β_*i*_ represents the linear effect of *x_i_*, β_*ij*_ represents the interaction between *x_i_* and *x_j_*, and β_*ii*_ represents the quadratic effect of *x_i_*.

The analysis of variance (ANOVA) was assessed by analyzing the model, whose quality could be further confirmed by the coefficient of determination (*R*^2^). The statistical significance was tested by the *F*-test, and the significance of regression coefficients was confirmed by the *t*-test.

### Validation of the Experimental Model

This model was confirmed with regard to all of the three variables. The maximum productions of oligosaccharides predicted by Design Expert 8.0.6 in six combinations of medium components were conducted in triplicates and then compared with the predicted values.

### Identification of Oligosaccharides Derived From *G. lemaneiformis*

Samples were withdrawn from the fermenter (Erlenmeyer flask) for analysis at regular intervals, and 50 mL of suspensions was collected by centrifugation (10,000 × *g*, 5 min), which was carried out in triplicate. The kind of oligosaccharides in the supernatant was identified by thin layer chromatography (TLC) and high-performance liquid chromatography (HPLC; Dionex, U-3000, United States) using a PA-100 anion exchange chromatography column (250 mm × 4 mm, CarboPac, Dionex, United States). The mobile phase was 100 mM NaOH and 150 mM NaAc. The mobile phase was filtered with a 0.22-μm microfiltration membrane before use. The sample injection volume was set to be 25 μL, and the column was eluted at 25°C with a flow rate of 0.25 mL/min using a pulse ampere detector (PAD).

Pure neoagarobiose, agarotriose, neoagarotetraose, agaropentaose, neoagarohexaose, agaroheptaose, neoagarooctaose, agarononaose, neoagarodecaose, agaroundecaose, and neoagarododecaose (Qingdao BZ Oligo Biotech Co., Ltd., China) were used as reference standards in the TLC experiment. Pure neoagarobiose, agarotriose, neoagarotetraose, agaropentaose, and neoagarohexaose (Qingdao BZ Oligo Biotech Co., Ltd., China) were used as reference standards in HPLC.

### Statistical Analyses

All experiments were conducted in triplicates. Each value in the tables and figures was presented as the mean ± standard error (*n* = 3). Statistical analyses of the tested data were conducted using the *t*-test.

## Results

### Screening and Identification of Agarolytic Strains

**FIGURE 1:**
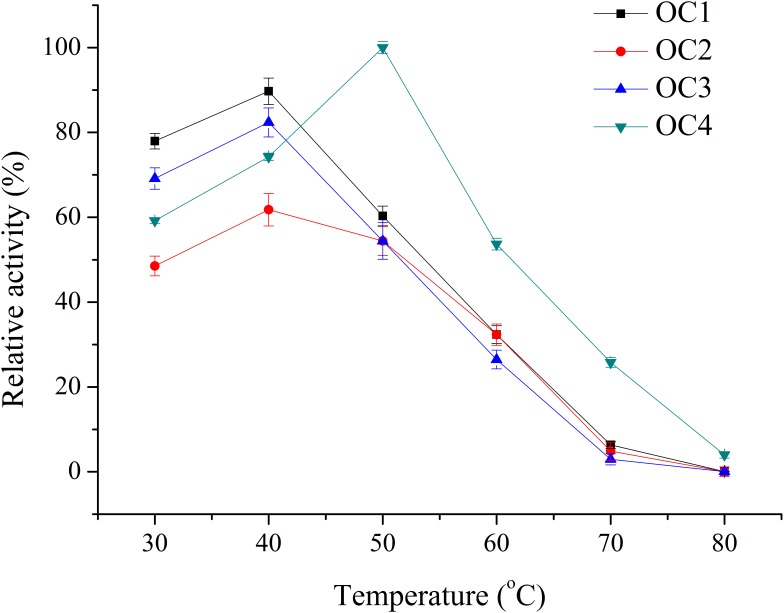
Temperature effect on the activity of the crude enzyme solution.

Four bacterial strains with transparent circle in the BCM plate were selected and named as OC1, OC2, OC3, and OC4. Crude enzyme of these strains was detected in the shaking flasks. The optimum temperature of the crude enzyme solution of OC4 was 50°C, while it was 40°C for OC1, OC2, and OC3. The relative enzyme activity at the optimal temperature of the agarolytic strains was OC4>OC1>OC3>OC2 (Figure 1). Thus, OC4 was selected for further research.

**FIGURE 2:**
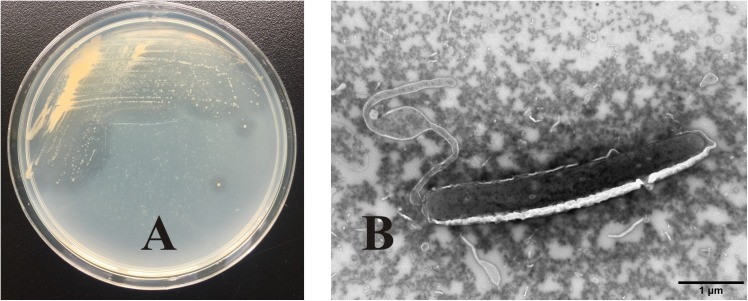
Colony morphology and cell morphology identification of strain OC4. **(A)** Colony morphology. **(B)** Cell morphology.

OC4 formed small yellowish round colonies, which were lustrous, regular, and easy to be picked up. There was a clear transparent circle around the colony, indicating that the secreted agarase degraded the agar in the medium (Figure 2A). TEM images showed that the strain cells with flagellum at the end of the tail were straight rod like, about 4 μm long and 0.6 μm wide (Figure 2B).

**FIGURE 3:**
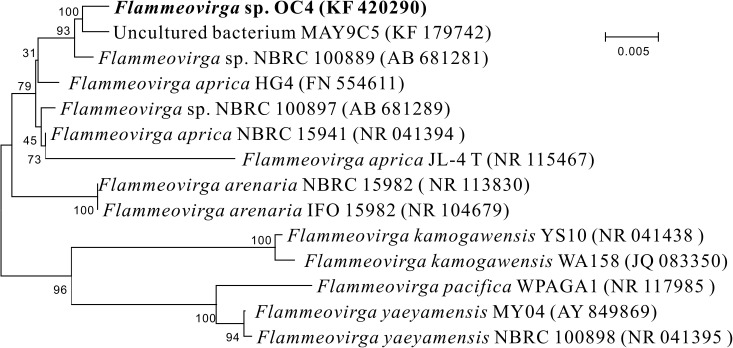
Neighbor-joining phylogenetic tree based on 16S rRNA gene sequences showing the positions of *Flammeovirga* sp. OC4 and representatives of related taxa. Bootstrap values based on 100 replications are shown at branch nodes. The scale bar indicates the average number of substitutions per site.

The 16S rRNA gene sequence of strain OC4 was acquired and then submitted to GenBank with the accession number KF 420290. Sequences of related taxa were obtained from GenBank using the program BLAST, and strains with the top hits are listed in Supplementary Table S1. Phylogenetic analysis showed that the closest relative for the new isolate was *Flammeovirga aprica* strain JL-4 with 98.07% 16S rRNA gene similarity. According to the position in the phylogenetic tree (Figure 3), this strain was named as *Flammeovirga* sp. strain OC4 and deposited in the Marine Culture Collection of China (MCCC) with accession number MCCC 1A07090.

### Characterization of Crude Agarase Enzyme

**FIGURE 4:**
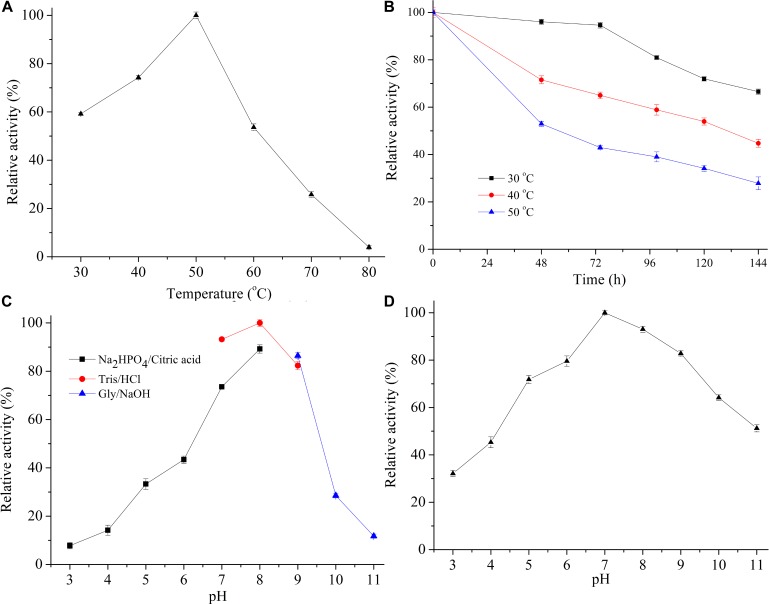
Effect of temperature and pH on enzyme activity of *Flammeovirga* sp. OC4. **(A)** The effect of temperature on enzyme activity. The activity was detected at the temperature ranges from 30 to 80°C at pH 7.4. **(B)** The effect of temperature on the stability of the enzyme. The remaining activity was measured after incubating crude enzyme in the absence of substrate at 30°C, 40°C, and 50°C for various durations. **(C)** pH effects on the activity of crude enzyme. pH profiles were determined by incubating crude enzyme at 50°C in the following buffers: Na2HPO4/citric acid solution (pH 3.0–8.0), Tris-HCl buffer (pH 7.0–9.0), and Gly/NaOH buffer (pH 9.0–11.0). **(D)** pH effects on the stability of crude enzyme. Prior to determination of residual activity, the crude enzyme was first incubated in buffers of desired pH (pH 3.0–11.0) at 50°C for 60 min. For all of the above plots, values are presented as percentages of the maximum activity of crude enzyme (taken as 100%) and are expressed as mean of three parallel trials with standard deviation.

The effect of temperature on the crude enzyme activity of *Flammeovirga* sp. OC4 was conducted at a wide temperature range (30–80°C) (Figure 4A). The optimum temperature was determined at 50°C, at which the enzyme had maximum activity and was set as 100%. The crude enzyme of *Flammeovirga* sp. OC4 retained 45% and 28% after incubation at 40°C and 50°C for 144 h, respectively (Figure 4B). The pH profile showed that the crude enzyme of *Flammeovirga* sp. OC4 had its optimum pH at 8.0, with more than 40% of its maximum activity in the spectrum from pH 6.0 to 9.0 (Figure 4C), and had more than 60% of the maximum activity after incubating for 60 min at pH 5.0–10.0 (Figure 4D).

The effects of EDTA and several metal ions on the activity of *Flammeovirga* sp. OC4 were investigated and listed in Table 2. Among these metal ions, Mn^2+^ and Ba^2+^ promoted the activity of *Flammeovirga* sp. OC4 especially. Mn^2+^ obviously enhanced the activity of *Flammeovirga* sp. OC4 by more than 14%. However, other metal ions (K^+^, Na^+^, Mg^2+^, Sr^2+^, Fe^2+^, Co^2+^, Zn^2+^, Ca^2+^, and Cu^2+^) exerted inhibitory effects on *Flammeovirga* sp. OC4 activity. Moreover, EDTA distinctly restrained the activity of the crude enzyme, deducing the important roles of certain divalent metal ions in the *Flammeovirga* sp. OC4 activity.

**Table 2:** Effects of metal ions and chemical agents on the enzyme activity.

Metal ion (10 mM)	Relative activity (%)	Metal ion (10 mM)	Relative activity (%)
K^+^	58.49 ± 2.73	Fe^2+^	92.02 ± 4.03
Na^+^	55.03 ± 3.05	Zn^2+^	48.38 ± 1.89
Sr^2+^	94.91 ± 1.95	Ba^2+^	107.83 ± 2.33
Mg^2+^	74.56 ± 4.16	Ca^2+^	60.23 ± 3.21
Cu^2+^	40.57 ± 3.17	Co^2+^	96.35 ± 2.45
Mn^2+^	114.56 ± 2.56	EDTA	51.56 ± 1.68
Control	100		

### Effects of Each Single Factor on the Production of Oligosaccharides

**FIGURE 5:**
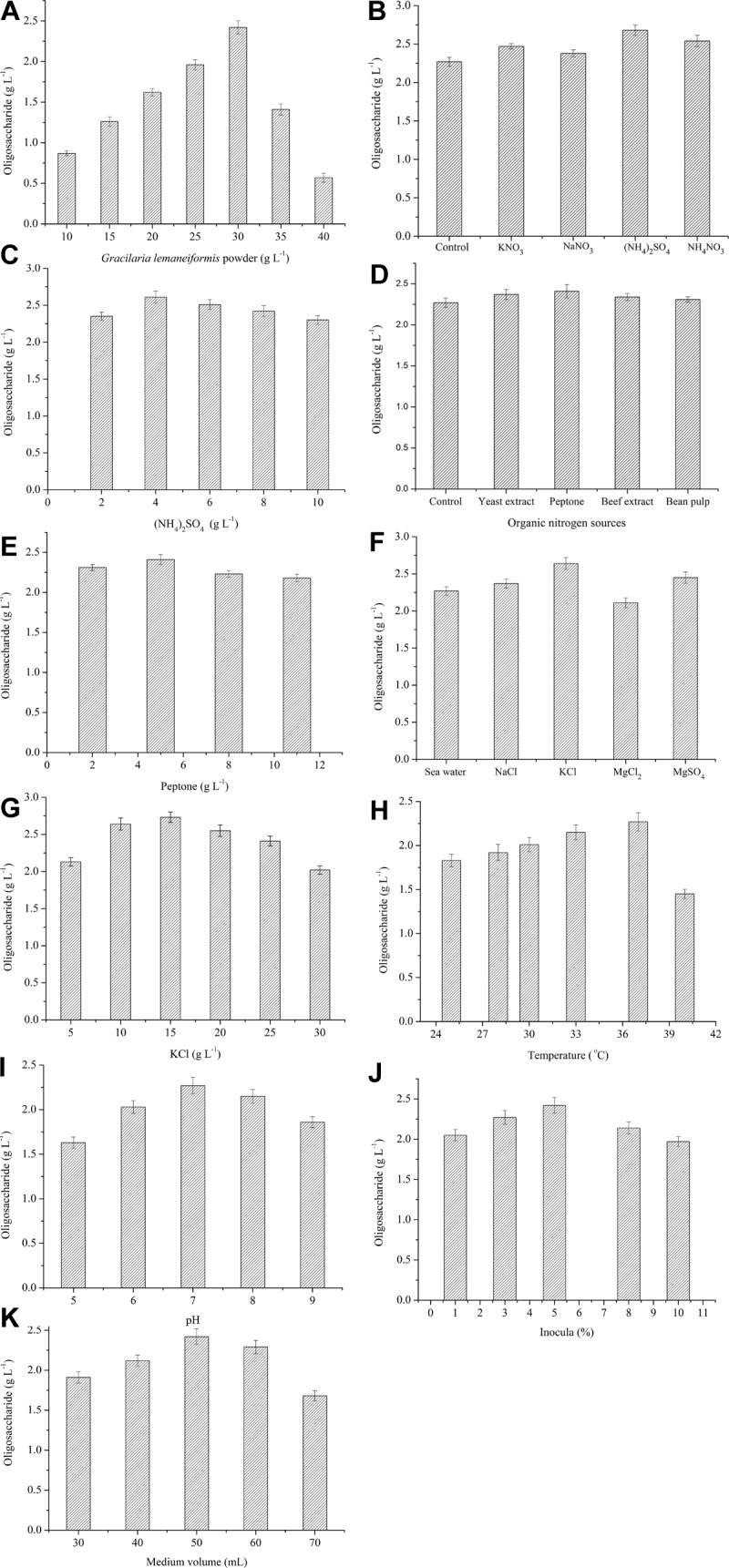
Effects of each single variable on the production of oligosaccharides. **(A)** Effect of *G. lemaneiformis*. The concentration was from 10 to 40 g L^−1^. **(B)** Effect of inorganic nitrogen sources. 5 g L^−1^ of KNO_3_, NaNO_3_, (NH_4_)_2_SO_4_, NH4NO_3_ were tested. **(C)** Effect of (NH_3_)_2_SO_4_. The concentration of (NH_4_)_2_SO_4_ was from 2 to 10 g L^−1^. **(D)** Effect of organic nitrogen sources. 4 g L^−1^ of yeast extract, peptone, beef extract, bean pulp were tested. **(E)** Effect of peptone. The concentration of peptone was from 2 to 11 g L^−1^. **(F)** Effect of inorganic salts. 10 g L^−1^ of NaCl, KCl, MgCl_2_, MgSO_4_ and sea water were tested. **(G)** Effect of KCl. The concentration of KCl was from 5 to 30 g L^−1^. **(H)** Effect of temperature. Different temperatures (25, 28, 30, 33, 37, and 40°C) were tested. **(I)** Effect of initial pH. Different initial pH (5, 6, 7, 8, 9) were tested. **(J)** Effect of inocula. Different inocula (1%, 3%, 5%, 8%, 10%) were tested. **(K)** Effect of medium volume. Different medium volume (30, 40, 50, 60, and 70 mL) were tested.

The production of oligosaccharides by *Flammeovirga* sp. OC4 in different conditions was measured, respectively. To evaluate the effect of the concentration of *G. lemaneiformis* on the production of oligosaccharides, the fermentation experiments were carried out at 37°C for 42 h using various amounts of *G. lemaneiformis*. The results show that the production of oligosaccharides increased from 0.86 to 2.27 g L^−1^ with increase in the concentration of *G. lemaneiformis* from 10 to 30 g L^−1^ (Figure 5A). However, when the concentration of *G. lemaneiformis* was higher than 30 g L^−1^, the production of oligosaccharides decreased (Figure 5A), which illustrated that a suitable concentration of *G. lemaneiformis* was beneficial for producing oligosaccharides. A high concentration of *G. lemaneiformis* in the fermentation could remarkably increase the viscosity of the culture, which might limit the oxygen and mass transfer and, thus, result in greatly reducing the production of oligosaccharides. Based on the above results, the concentration of *G. lemaneiformis* (30 g L^−1^) was used as the carbon source concentration in the optimization of the fermentation process.

Both organic and inorganic nitrogen sources have influence on the production of oligosaccharides from *G. lemaneiformis* by the action of *Flammeovirga* sp. OC4. As shown in Figure 5B,D, (NH_4_)_2_SO_4_ and peptone were the most suitable inorganic and organic nitrogen source. The maximum concentration of oligosaccharide was observed at 4 g L^−1^ (NH_4_)_2_SO_4_ and 5 g L^−1^ peptone (Figure 5C,E). It means that suitable nitrogen sources can accelerate the growth of *Flammeovirga* sp. OC4, which increased the production of oligosaccharides, whereas the high concentration of nitrogen sources made *Flammeovirga* sp. OC4 grow too fast, thus inhibiting its degrading activity on *G. lemaneiformis*.

Since seawater was difficult to get inland on a large scale, the impact of inorganic salt water solution on the production of oligosaccharides was studied. As shown in Figure 5F, the highest production of oligosaccharides was observed in KCl solution compared to seawater, NaCl, MgSO_4_, and MgCl_2_ solution. The optimal concentration of KCl was found to be 15 g L^−1^ (Figure 5G).

The effects of different temperatures, initial pH, inocula, and working medium volume on the production of oligosaccharides from the biotransformation of *G. lemaneiformis* by the action of *Flammeovirga* sp. OC4 are shown in Figure 5H,I,K. The highest production of oligosaccharides among different temperatures, initial pH, inocula, and working medium volume were observed at 37°C (Figure 5H), pH 7 (Figure 5I), 5% inocula (Figure 5J), and 50-mL working medium volume (Figure 5K), respectively.

### Screening of Significant Factors by the PB Design

To increase the yield of oligosaccharides, seven medium components were screened by the PB design, which contained 12 experimental runs and two levels for each component. The effects of each medium component on the yield of oligosaccharides are summarized in Table 3.

**Table 3:** PB experimental design for evaluation of 12 components with coded values for oligosaccharide production by *Flammeovirga* sp. OC4.

Run	A	B	D	E	G	H	I	K	Oligosaccharides (g L^−1^)
1	1	1	1	1	−1	−1	−1	−1	2.42 ± 0.09^∗^
2	−1	1	−1	1	1	−1	−1	1	2.31 ± 0.07^∗^
3	1	−1	1	−1	1	1	−1	−1	1.92 ± 0.06^∗^
4	−1	1	1	1	1	1	1	−1	1.99 ± 0.08^∗^
5	−1	−1	−1	1	−1	1	1	−1	2.17 ± 0.08^∗^
6	−1	−1	1	−1	1	−1	1	1	2.45 ± 0.10^∗^
7	1	−1	−1	1	1	1	−1	1	2.30 ± 0.01^∗^
8	1	1	−1	−1	−1	1	1	1	2.06 ± 0.13^∗^
9	1	1	−1	−1	1	−1	1	−1	2.71 ± 0.12^∗^
10	−1	1	1	−1	−1	1	−1	1	2.18 ± 0.67^∗^
11	1	−1	1	1	−1	−1	1	1	2.25 ± 0.09^∗^
12	−1	−1	−1	−1	−1	−1	−1	−1	2.36 ± 0.11^∗^

*All experiments were conducted in triplicates. ^∗^Statistical analysis by t-test, P < 0.05.*

**FIGURE 6:**
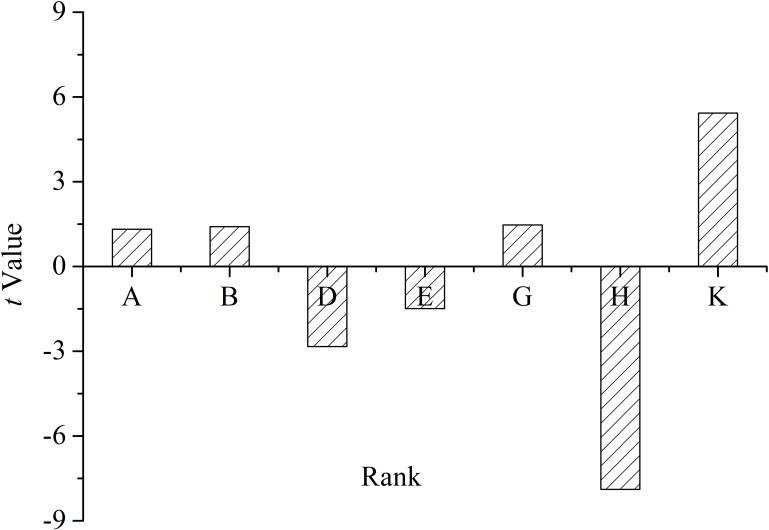
*T*-value of the medium constituents for oligosaccharide production by *Flammeovirga* sp. OC4 based on the Plackett–Burman (PB) experimental results. (A) Peptone; (B) KCl; (D) (NH_4_)_2_SO_4_; (E) initial pH; (G) inocula; (H) working medium volume; and (K) temperature.

The *t*-value on the effect of each medium component is shown in Figure 6, which reflected the contributions of each component to the yield of oligosaccharides.

The medium components influencing the yield of oligosaccharides were ranked as follows: working medium volume > temperature > (NH_4_)_2_SO_4_ > inocula > initial pH > KCl > peptone. It was clear that working medium volume, initial pH, and (NH_4_)_2_SO_4_ affected oligosaccharide production in a negative way, and temperature, inocula, KCl, and peptone affected the yield in a positive way (Figure 6). According to the PB design, the highest oligosaccharide production (2.71 g L^−1^) was found in run 9, while the lowest yield (1.92 g L^−1^) was in run 3 (Table 3).

From these experimental runs, the standard ANOVA results were reckoned (Supplementary Table S2). The *F*-value (14.76) and *P*-value (0.0103) of the model indicate that the model and its terms were significant. Based on the ANOVA results, working medium volume, temperature, and (NH_4_)_2_SO_4_ were selected as significant model terms. Moreover, the value of *R*^2^ for the model was 0.9627. The predicted *R*^2^ values corresponded with the adjusted *R*^2^ values for all equations. The adequate precision that measures the signal-to-noise ratio was 12.768 for oligosaccharide production. A desirable ratio of >4 was recorded in this study. According to the results, the three most significant variables [working medium volume, temperature, and (NH_4_)_2_SO_4_] were selected for further study.

### Optimization Using RSM

As the highest oligosaccharide production was found in run 9, their level was determined as the center points. The optimal condition for the three significant factors was determined using CCD under RSM. One of the 20 predicted combinations from the regression equation was set in each run, and the experimental oligosaccharide production in each run was obtained (Table 4). It was found that the maximal yield of oligosaccharides (3.14 g L^−1^) by *Flammeovirga* sp. OC4 was in run 7, while run 10 showed the minimum production (2.75 g L^−1^).

**Table 4:** Experimental design using CCD of three independent variables with their actual and coded values and six center points showing the experimental and predicted responses.

Run	Factors			Oligosaccharides (g L^−1^)
	Medium volume (mL)	Temperature (°C)	(NH_4_)_2_SO_4_ (g L^−1^)	Experimental
1	40 (−1)	33 (−1)	2 (−1)	2.99 ± 0.14^∗^
2	60 (1)	33 (−1)	2 (−1)	2.75 ± 0.09^∗^
3	40 (−1)	37 (1)	2 (−)	3.10 ± 0.11^∗^
4	60 (1)	37 (1)	2 (−1)	2.92 ± 0.15^∗^
5	40 (−1)	33 (−1)	6 (1)	2.94 ± 0.08^∗^
6	60 (1)	33 (−1)	6 (1)	2.78 ± 0.09^∗^
7	40 (−1)	37 (1)	6 (1)	3.14 ± 0.11^∗^
8	60 (1)	37 (1)	6 (1)	2.97 ± 0.12^∗^
9	26.4 (−1.682)	35 (0)	4 (0)	3.03 ± 0.09^∗^
10	73.6 (1.682)	35 (0)	4 (0)	2.75 ± 0.08^∗^
11	50 (0)	31.6 (−1.682)	4 (0)	2.82 ± 0.08^∗^
12	50 (0)	38.4 (1.682)	4 (0)	3.11 ± 0.12^∗^
13	50 (0)	35 (0)	0.636 (−1.682)	3.03 ± 0.16^∗^
14	50 (0)	35 (0)	7.364 (1.682)	3.07 ± 0.10^∗^
15	50 (0)	35 (0)	4 (0)	3.09 ± 0.10^∗^
16	50 (0)	35 (0)	4 (0)	3.11 ± 0.11^∗^
17	50 (0)	35 (0)	4 (0)	3.08 ± 0.11^∗^
18	50 (0)	35 (0)	4 (0)	3.11 ± 0.10^∗^
19	50 (0)	35 (0)	4 (0)	3.13 ± 0.10^∗^
20	50 (0)	35 (0)	4 (0)	3.09 ± 0.09^∗^

*All experiments were conducted in triplicates. ^∗^Statistical analysis by t-test, P < 0.05. CCD, central composite design.*

The regression equation below displayed the dependence of the yield of oligosaccharides on constituents of the medium.

$$\begin{array}{c} \text{Y}({\text{oligosaccharides, gL}}^{-1})=3.10-0.89\text{A}+0.084\text{B}\\ \;+9.319{\text{E}}^{-003}\text{C}+5.000{\text{E}}^{-003}\text{AB}+0.01\text{AC}\\ \;+0.015\text{BC}-0.077{\text{A}}^{2-}0.05{\text{B}}^{2}-0.02{\text{C}}^{2} \end{array}$$

The response (Y) represented the yield of oligosaccharides, and A, B, and C, respectively, represented the working medium volume, temperature, and (NH_4_)_2_SO_4_.

Supplementary Table S3 presented the ANOVA results for the quadratic model. The *P*-value of the model was less than 0.05, which implied that the model was significant. In this case, A, B, C, AC, BC, A^2^, B^2^, and C^2^ were significant model terms. The lack of fit of *P* = 0.047 shows that the lack of fit was significant. These results reveal that the second-order equation could be used to predict the effects of the variables [working medium volume, temperature, and (NH_4_)_2_SO_4_] on the yield of oligosaccharides by *Flammeovirga* sp. OC4 in shaking Erlenmeyer flask cultures.

The coefficient values of the regression equation are also listed in Supplementary Table S3. *R*^2^ close to 1 meant that the correlation between the predicted values and experiment results was high.

**FIGURE 7:**
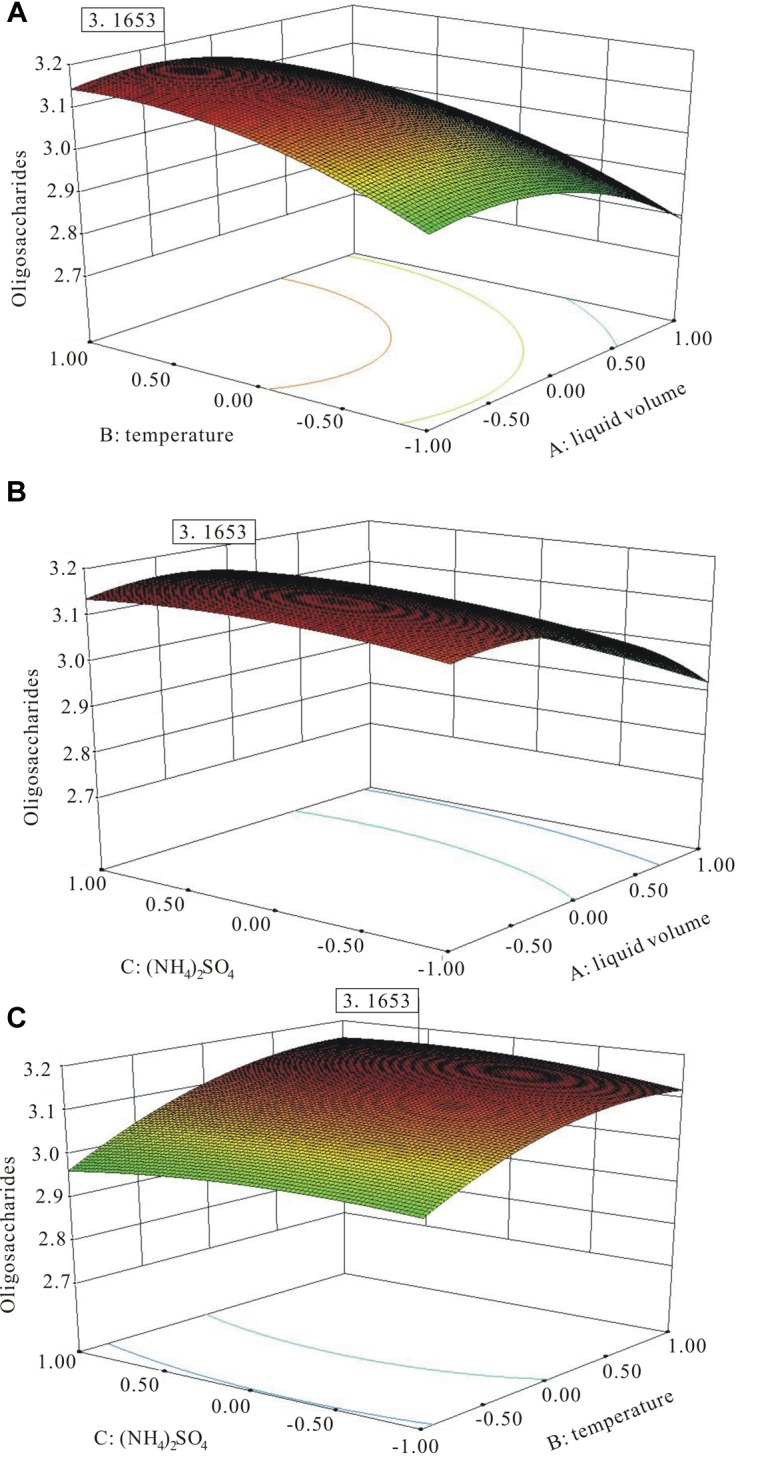
The three-dimensional (3D) response surface curves of the combined effects of medium volume, temperature, and (NH_4_)_2_SO_4_ on oligosaccharide production. **(A)** Medium volume and temperature at the fixed level of (NH_4_)_2_SO_4_. **(B)** Medium volume and (NH_4_)_2_SO_4_ at the fixed level of temperature. **(C)** (NH_4_)_2_SO_4_ and temperature at the fixed level of medium volume.

The three-dimensional (3D) response surface plots (Figure 7) were the graphical representation of the regression equation, which indicated the following predicted optimal fermentation condition: *A* = −0.52, *B* = 0.87, and *C* = 0.42, corresponding to the optimal levels of 44.8 mL working medium volume, 36.7°C temperature, and 4.84 g L^−1^ (NH_4_)_2_SO_4_ for the maximal oligosaccharide production of 3.16 g L^−1^.

### Validation of the Model

To test the accuracy of the predicted results, several experiments were replicated three times, and the medium components are presented in Table 5. Some of the experimental results are slightly lower or higher than the predicted results, but all of the deviations are smaller than 5%. This might be due to unavoidable experimental errors.

**Table 5:** Validation of the experimental model.

Experiment	Medium volume (mL)	Temperature (°C)	(NH_4_)_2_SO_4_ (g L^−1^)	Oligosaccharides (g L^−1^)	
				Experimental	Predicted
1	46.6	34.7	2.08	3.02 ± 0.075^∗^	3.00
2	35.7	33.2	1.56	2.98 ± 0.082^∗^	3.13
3	39.6	32.6	3.20	3.01 ± 0.102^∗^	3.08
4	44.8	36.7	4.84	3.09 ± 0.087^∗^	3.16
5	32.1	32.9	3.70	3.03 ± 0.093^∗^	3.11

*All experiments were conducted in triplicates. ^∗^Statistical analysis by t-test, P < 0.05.*

After optimization, a 36.1% increase in oligosaccharide production to 3.09 g L^−1^ was observed with fermentation performed using CCD, which was compared with the oligosaccharide production (2.27 g L^−1^) obtained using the original culture medium.

### Identification of Oligosaccharides Derived From *G. lemaneiformis*

**FIGURE 8:**
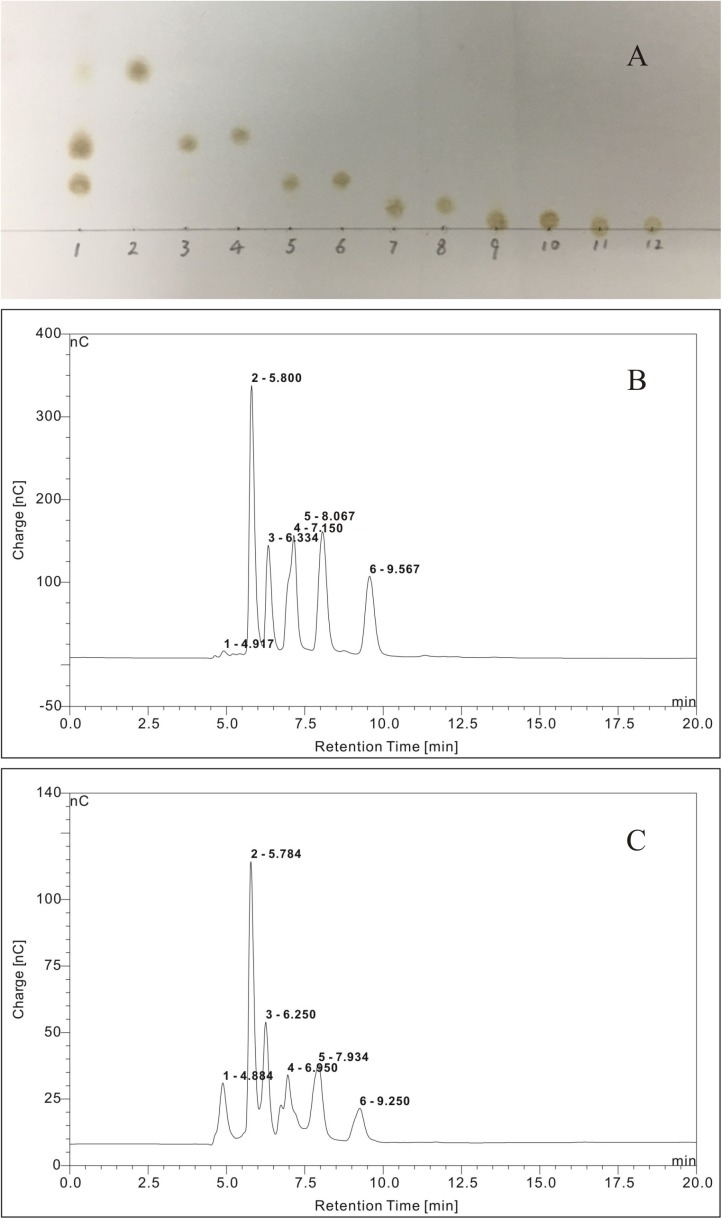
Thin layer chromatography (TLC) analysis **(A)** and high-performance liquid chromatography (HPLC) chromatograms **(B,C)** of purified fermentation products of *Flammeovirga* sp. OC4. **(A)** Lane 1, purified fermentation products; lane 2, neoagarobiose; lane 3, agarotriose; lane 4, neoagarotetraose; lane 5, agaropentaose; lane 6, neoagarohexaose; lane 7, agaroheptaose; lane 8, neoagarooctaose; lane 9, agarononaose; lane 10, neoagarodecaose; lane 11, agaroundecaose; and lane 12, neoagarododecaose.

The TLC chromatogram of the reference standards of oligosaccharides and oligosaccharides derived from *G. lemaneiformis* is displayed in Figure 8A. It could be deduced that the oligosaccharides derived from *G. lemaneiformis* were agarotriose, agaropentaose, neoagarobiose, neoagarotetraose, and neoagarohexaose. The HPLC chromatograms, which are displayed in Figure 8B,C, respectively, further confirmed this deduction. Peaks 1, 2, 3, 4, and 5 in Figure 8B represented the peak of reference substance of agarotriose, agaropentaose, neoagarobiose, neoagarotetraose, and neoagarohexaose, respectively. A comparison to the HPLC chromatogram of the reference standards of oligosaccharides showed that there were the same peaks 1, 2, 3, 4, and 5 in the HPLC chromatogram of the oligosaccharides derived from *G. lemaneiformis* (Figure 8C), indicating that the oligosaccharides derived from *G. lemaneiformis* in this work contained agarotriose, agaropentaose, neoagarobiose, neoagarotetraose, and neoagarohexaose.

## Discussion

*In situ* enrichment was beneficial for screening target strains. According to the traditional method, samples collected in the deep sea were treated in the laboratory; thus, screening of target strains was challenged by both the randomness of sampling and the change of habitats. In the separation process, if the target strain did not have absolute competitive edge, it was likely to submerge in a large number of other strains. However, *in situ* enrichment created a favorable habitat for target strains, which still circulated in the environment of the deep sea. After a period of time, the target strain will occupy the advantage to facilitate our subsequent screening. In this study, four strains with agar-degrading activity were screened without much interruption from other irrelevant strains.

Compared with traditional methods of producing algae oligosaccharides from algae polysaccharides, the “one-step process” established in this study had at least two advantages. The first one is the reduction in the pollutants (i.e., by-products) of algal dregs in the extraction process of agar from *G. lemaneiformis*. Algal dregs contain diatomite and perlite, which were added in the extraction process to facilitate filtering of agar, and a high concentration of salt produced by acid–base neutralization. The dregs would obstruct the growth of plants, crops, fish, and shrimps, causing dead corners of environmental sanitation if they were accumulated in large quantities. The second one is the retaining of the beneficial ingredients of algae. It is mentioned above that algae oligosaccharides are the degradation products of the cell walls of algae. In addition to cell walls, algae also have many beneficial components, such as algae proteins, polyterpenoids, and steroids, which have the potential of promoting growth of plants and crops and would be destroyed by strong acid and alkali. In this study, oligosaccharides with different degrees of polymerization were produced by “one-step process,” thus retaining beneficial ingredients without producing dregs.

Response surface methodology effectively combined mathematical methods with statistical analysis. Through mathematical modeling and analysis of several response process variables, the optimal conditions of a multifactor system could be determined efficiently. Compared with the orthogonal experiment method, RSM could make a comprehensive study of the experiment with less experiment quantity and time and scientifically provide the relationship between the whole and the partial, which could obtain more definite and effective conclusions. In this study, three factors that had the greatest influence on the number of oligosaccharides produced by the “one-step process” were determined by the PB test. After optimization of RSM, the yield of oligosaccharides was increased by 36.1%, which was from 2.71 to 3.09 g L^−1^. The results demonstrated that the “one-step process” had potential for large-scale industrial applications.

## Conclusion

A “one-step process” for the preparation of oligosaccharides from *G. lemaneiformis* was established through acquisition of *Flammeovirga* sp. OC4 by *in situ* enrichment in the deep sea and the following optimization methods. This study provided an alternative approach for environment-friendly and sustainable utilization of algae on a large scale. In addition to creating more opportunities for the application of *G. lemaneiformis* in the industry, the “one-step process” described in this study could be extended to other kinds of algae in the future.

## Author Contributions

HL and RZ designed the research program. XC, LL, and ZC performed the experiments. XC analyzed the experimental data and wrote the manuscript. HL revised the manuscript and ML edited the English language of the manuscript. All authors have approved the submission and publication of the manuscript.

## Conflict of Interest Statement

The authors declare that the research was conducted in the absence of any commercial or financial relationships that could be construed as a potential conflict of interest.
