# Supplementary figures and images for: One-Step Process for Environment-Friendly Preparation of Agar Oligosaccharides From Gracilaria lemaneiformis by the Action of Flammeovirga sp. OC4
Source: Front Microbiol. 2019 Apr 17;10:724. doi: 10.3389/fmicb.2019.00724 (PMC6478668; doi:10.3389/fmicb.2019.00724)

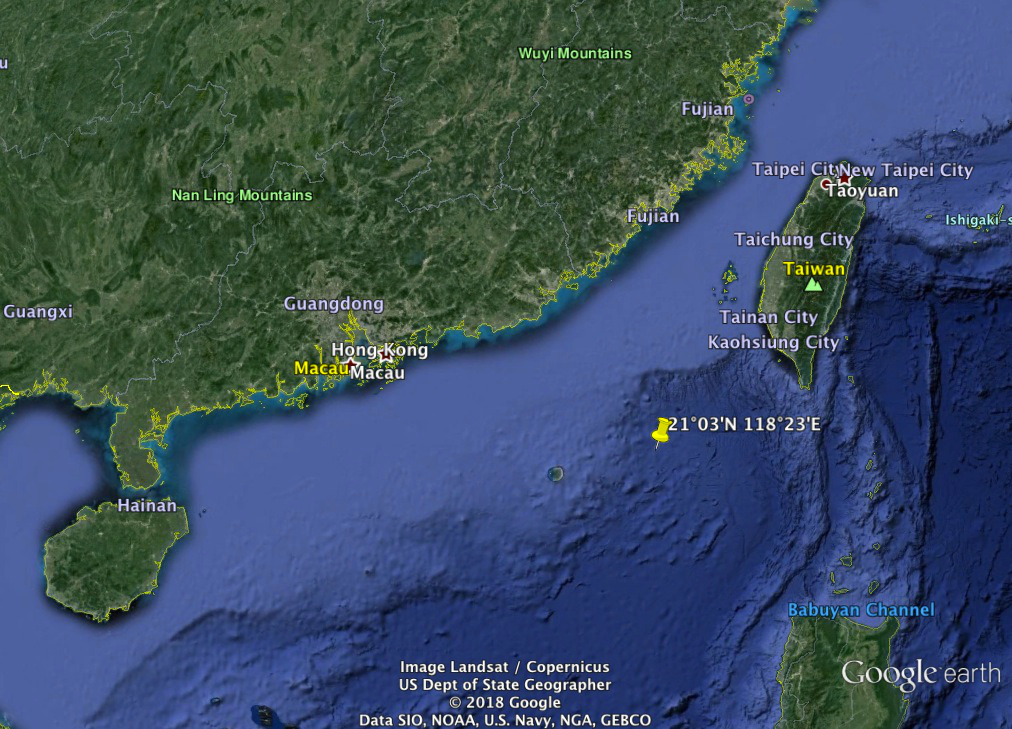

Supplement: Supplementary file 1 [file Image_1.tif]
