# Supplementary material for: One-Step Process for Environment-Friendly Preparation of Agar Oligosaccharides From Gracilaria lemaneiformis by the Action of Flammeovirga sp. OC4
Source: Front Microbiol. 2019 Apr 17;10:724. doi: 10.3389/fmicb.2019.00724 (PMC6478668; doi:10.3389/fmicb.2019.00724)
Supplement: Supplementary file 2 [file Table_1.docx]

**TABLE S1** Strains top hit to *Flammeovirga* sp. OC4

| Strains | Identity | |
| --- | --- | --- |
| Uncultured bacterium clone MAY9C5 | | 99% |
| *Flammeovirga* sp. NBRC 100889 | | 99% |
| *Flammeovirga* sp. NBRC 100897 | | 99% |
| *Flammeovirga aprica* HG4 | | 98% |
| *Flammeovirga aprica* strain NBRC 15941 | | 98% |
| *Flammeovirga arenaria* strain NBRC 15982 | | 97% |
| *Flammeovirga arenaria* strain IFO 15982 | | 97% |
| *Flammeovirga yaeyamensis* strain MY04 | | 95% |
| *Flammeovirga pacifica* strain WPAGA1 | | 95% |
| *Flammeovirga yaeyamensis* strain NBRC 100898 | | 95% |
| *Flammeovirga kamogawensis* strain YS10 | | 95% |
| *Flammeovirga kamogawensis* strain WA158 | | 94% |
| *Flammeovirga aprica* strain JL-4 | | 92% |

**TABLE S2** The statistical analysis of medium components for oligosaccharides production by *Flammeovirga* sp. OC4 using the PB design

| Variable | | | Sum of squares | Coefficient estimate | Mean squares | Standardized  effects | *F* value | *P* |
| --- | --- | --- | --- | --- | --- | --- | --- | --- |
| Code | | Factors |  |  |  |  |  |  |
| Model | |  | 0.50 | 2.26 | 0.072 |  | 14.76 | 0.0103 ^a^ |
| A | peptone | | 3.333E-003 | 0.017 | 3.333E-003 | 0.033 | 0.69 | 0.4541 |
| B | KCl | | 4.033E-003 | 0.018 | 4.033E-003 | 0.037 | 0.83 | 0.4138 |
| D | (NH_4_)_2_SO_4_ | | 0.041 | -0.058 | 0.041 | -0.120 | 8.40 | 0.0442* |
| E | initial pH | | 4.800E-003 | -0.020 | 4.800E-003 | -0.040 | 0.99 | 0.3765 |
| G | inocula | | 4.800E-003 | 0.020 | 4.800E-003 | 0.040 | 0.99 | 0.3765 |
| H | medium volume | | 0.29 | -0.16 | 0.29 | -0.31 | 60.62 | 0.0015* |
| K | temperature | | 0.15 | 0.11 | 0.15 | 0.22 | 30.80 | 0.0052* |

^a^ *P* <0.05 means significant, the symbol * shows significant difference.

**TABLE** **S3** ANOVA results for the quadratic model

| Source | Sum of  square | df | Coefficient estimate | Mean squares | *F* value | *P* |
| --- | --- | --- | --- | --- | --- | --- |
| Model ^a^ | 0.32 | 9 | 3.10 | 0.036 | 124.66 | < 0.0001 |
| A−A | 0.11 | 1 | -0.089 | 0.11 | 375.12 | < 0.0001 |
| B−B | 0.096 | 1 | 0.084 | 0.096 | 337.00 | < 0.0001 |
| C−C | 1.186E-003 | 1 | 9.319E-003 | 1.186E-003 | 4.14 | 0.0491 |
| AB | 2.000E-004 | 1 | 5.000E-003 | 2.000E-004 | 0.70 | 0.4227 |
| AC | 8.000E-004 | 1 | 0.010 | 8.000E-004 | 2.80 | 0.1255 |
| BC | 1.800E-003 | 1 | 0.015 | 1.800E-003 | 6.29 | 0.0310 |
| A^2^ | 0.085 | 1 | -0.077 | 0.085 | 298.51 | < 0.0001 |
| B^2^ | 0.037 | 1 | -0.050 | 0.037 | 128.31 | < 0.0001 |
| C^2^ | 6.014E-003 | 1 | -0.020 | 6.014E-003 | 21.01 | 0.0010 |
| Residual | 2.862E-003 | 10 |  | 2.862E-004 |  |  |
| Lack of Fit | 1.179E-003 | 5 |  | 2.358E-004 | 0.70 | 0.047 |
| Pure Error | 1.683E-003 | 5 |  | 3.367E-004 |  |  |
| Cor Total | 0.32 | 19 |  |  |  |  |

^a^ R−Squared =0.9869, Adj R−Squared =0.9751, CV=2.59, Adeq Precision ratio=24.855
